# FOOD INSECURITY AND CANCER SCREENING BEHAVIORS AMONG BLACK MEN LIVING IN FOOD DESERTS

**DOI:** 10.1093/geroni/igae098.1598

**Published:** 2024-12-31

**Authors:** Darlingtina Esiaka, Obinna Odo, Kingsley Udeh

**Affiliations:** University of Kentucky College of Medicine, University of Kentucky, Kentucky, United States; Miami University, Oxford, Ohio, United States; Miami University, Oxford, Ohio, United States

## Abstract

Research shows an intricate relationship between food insecurity and health behaviors. The relationship reflects the profound impact of inadequate access to nutritious food on overall well-being. Individuals facing food insecurity often encounter barriers to adopting health-promoting behaviors, influencing their physical health outcomes. This study investigated the relationship between food insecurity and cancer screening behaviors among Black men dwelling in food deserts, examining a critical intersection of health disparities. Participants (N=186; Mean age = 48.45, SD = 10.95) responded to questions assessing their demographic characteristics, experience with food insecurity, health risk behaviors, healthcare access, and current and future cancer screening behaviors. We found a significant association between food insecurity and current cancer screening behaviors (B=-.118, p=0.043) but not between food insecurity and future cancer screening behaviors (B=-.048, p=0.329). Black men experiencing food insecurity were less likely to engage in cancer screening compared to their counterparts with more reliable access to nutritious food. Also, we found that socioeconomic status (B=-.163, p=0.006), medical insurance (B=-.672, p=0.019), and access to primary care physician (B=-.609, p=0.026) were significantly associated with future cancer screening behaviors. Understanding the link between food insecurity and cancer screening behavior provides crucial insights for tailoring targeted interventions that consider the unique challenges faced by individuals living in food deserts, particularly within the context of racial and ethnic health disparities.

